# Phthalate Esters in Different Types of Cosmetic Products: A Five-Year Quality Control Survey

**DOI:** 10.3390/molecules29204823

**Published:** 2024-10-11

**Authors:** Natalia Aldegunde-Louzao, Manuel Lolo-Aira, Carlos Herrero-Latorre

**Affiliations:** 1 Aquatic One Health Research Center (ARCUS), Analytical Chemistry Nutrition and Bromatology Department, Faculty of Sciences, Universidade de Santiago de Compostela, Campus Terra, 27002 Lugo, Spain; natalia.aldegunde@rai.usc.es; 2 Applied Mass Spectrometry Laboratory (AMSlab), Rúa do Vidro, 117D, 27003 Lugo, Spain; manuel.lolo@ams-lab.com

**Keywords:** phthalate esters, determination, GC-MS/MS, cosmetics, quality control

## Abstract

Phthalate esters are commonly included in the formulations of cosmetics and related products in order to retain fragrance, enhance flexibility (i.e., by acting as plasticizers), facilitate the dissolution and dispersion of other ingredients, and improve the overall texture and sensory experience of the products. This study aimed to assess the presence and concentrations of phthalates in cosmetics by analyzing a comprehensive set of samples collected over a period of five years (2016–2020). The concentrations of nine different phthalate esters (BBP, DEHP, DNOP, DPP, DBP, DIPP, DMEP, DMP and PIPP) in 1110 cosmetics samples from France and Spain were determined by gas chromatography–mass spectrometry. The samples were included in five categories: soaps and shampoos; hand and body creams; lip gloss and lipsticks; nail polish; and facial makeup and skincare products. Some of the samples (4.86%) contained at least one phthalate at concentrations above the threshold limit (1 µg mL^−1^). Variable concentrations of different phthalates were determined in the 54 positive samples identified. DEHP was the most frequently detected phthalate, followed by DBP. The findings revealed different profiles according to the different categories of cosmetics and the phthalates detected in each. The results were critically compared with those obtained in various previous studies.

## 1. Introduction

**Figure 1:**
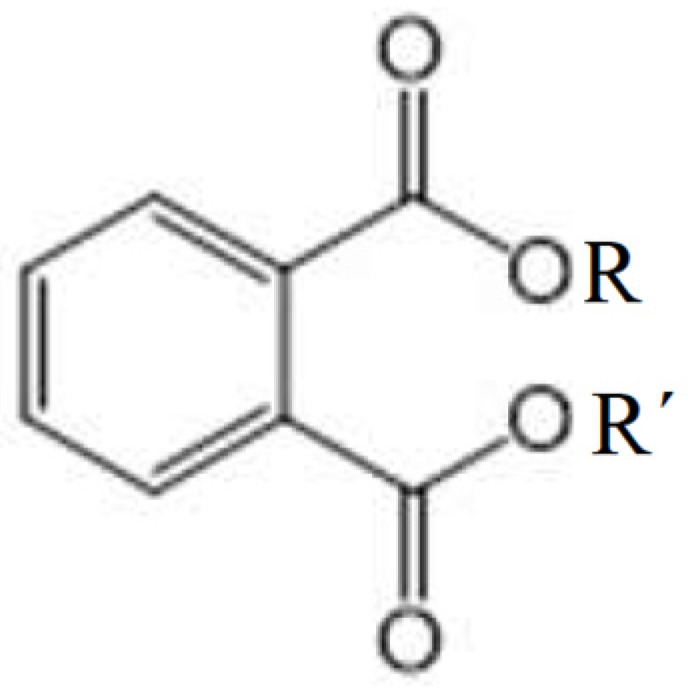
General structure of phthalates, where R and R’ are C_n_H_2n+1_ chains.

Phthalates or phthalate esters (PAEs) are the ortho-di-esters of phthalic acid with the general structure illustrated in Figure 1, where R and R’ represent C_n_H_2n+1_ chains. PAEs have been used for several decades as plasticizers in a variety of products [1]. Phthalates are included in different formulations of cosmetics for several purposes: (i) as fragrance fixatives, used to improve the stability and longevity of fragrances in cosmetics; (ii) as plasticizers (which make plastics more flexible and durable), used in products like nail polish to enhance flexibility and prevent cracking [2]; (iii) as solvents, helping to dissolve and disperse other ingredients in hairsprays and some nail care products; (iv) and finally, to enhance the texture and feel of cosmetics, increasing the spreadability, smoothness and overall sensory experience of the products [3,4]. In addition, PAEs can also migrate from plastics or other plasticized materials used to package the cosmetics, as well as from the packaging machinery and connected equipment, or from any other device that comes into contact with the products during the manufacturing process [5].

Despite the previously commented upon functional benefits, the use of phthalates in cosmetics has raised concerns because some studies suggest that there are potential health risks associated with these compounds [4,6]. Cosmetics are used for the main purposes of cleaning, perfuming, altering appearance, protecting or maintaining skin, and correcting body odors. They are therefore designed to come into close contact with the external layers of the human body (such as the epidermis, hair, nails, etc., as well as teeth and oral mucosa), which leads to direct exposure to phthalates contained in the formulations. Numerous studies have linked certain phthalates to endocrine disruption [7,8], reproductive toxicity [9,10] and other adverse health effects [11,12]. As a result, the governments of different countries have placed restrictions on the use of certain phthalates in cosmetics, implemented by various health agencies and regulatory bodies. However, although there is a recent and growing trend towards the development and consumption of cosmetics with phthalate-free formulations, in practice, phthalates continue to be widely used, and their control is essential.

Legislation on phthalates may vary depending on the country involved, but regulations in all countries have established limitations and maximum concentrations for certain phthalates in cosmetics. Labelling obligations are also imposed for these products, and the composition of the product must be declared. In the European Union, restricted phthalates and labelling requirements for cosmetics are detailed in EC Regulation 1223/2009 [13]. This law subjects phthalates to strict control, and several of these compounds have been banned as ingredients in cosmetics and personal care products due to possible carcinogenic and mutagenic effects on human health: dibutyl phthalate (DBP), bis(2-methoxyethyl) phthalate (DMEP), diisopentyl phthalate (DIPP), di-*n*-pentyl phthalate (DPP), benzyl butyl phthalate (BBP), bis(2-ethylhexyl) phthalate (DEHP), *n*-pentyl isopentyl phthalate (PIPP), di-*n*-octyl phthalate (DNOP) and dimethyl phthalate (DMP), among others. Moreover, the REACH [14] and CLP regulations [15] are also important in regard to controlling the use of phthalates. The first one regulates chemicals, including phthalates, requiring their registration, evaluation, authorization and restriction, ensuring the availability of safety data for contributing to the safe use of chemicals. On the other hand, the CLP regulation classifies substances as carcinogenic, mutagenic or toxic for reproduction (CMR) based on assessments by the European Chemicals Agency. CMR substances are generally banned in cosmetics by EC 1223/2009.

This paper presents the results of a five-year phthalate monitoring study (2016–2020) for cosmetics marketed in the European Union. A large sample of cosmetics from France and Spain, including shower gels, shampoo and soaps, hand and body creams and lotions, lipsticks, glitters and nail polishes, and facial make-up and skin cosmetics, were analyzed by gas chromatography to determine the presence and concentrations of nine different phthalates. The results obtained were critically compared with other published studies in which phthalates were determined in cosmetics.

## 2. Results

The cosmetics samples included in the five categories established were analyzed by GC-MS/MS to determine the concentrations of the nine phthalates following the procedure explained in Section 4.3. Legislation in Europe prohibits the presence of certain PAEs in cosmetics, but certainly, if highly sensitive analytical techniques are used (as is the case) and given the ubiquity of phthalates, they will be detected in most samples at extremely low concentrations. Since there is no regulated limit, the criteria used by the industry to consider a sample as positive (and therefore discard it or reprocess it before entering the market) depends on the different countries, the different manufacturers, and other social and health factors. The maximum limits allowed in other matrices, such as textiles, are around 1000 µg mL^−1^. In cosmetics, large manufacturers usually consider values between 1 and 5 µg mL^−1^. In this work, the lowest value of the interval (1 µg mL^−1^) was selected as the threshold from which the sample must be considered positive.

Phthalate esters were detected at concentrations above this threshold limit (1 µg mL^−1^) in only 54 of the 1110 samples measured. Thus, positive samples represented 4.86% of the total, which is a low but significant percentage. The distribution of positive samples according to the different categories is summarized in Table 1.

The percentage of positive samples was highest for category 2 (creams and body and hand lotions) and for category 3 (gloss and lipsticks), with 11.01 and 7.24% positive samples, respectively. This finding can be explained by considering that the addition of PAEs to this category of cosmetics enables fixation and enhancement of fragrance in lotions and creams and improves the absorption of other ingredients by the skin. Furthermore, the high percentage of positive samples in this category may also be due to the addition of PAEs with emollient properties to help to soften and moisturize the skin. In addition, these types of cosmetics are usually packaged in flexible plastic containers, which may lead to contamination of the product.

On the other hand, in category 3 cosmetics, the use of certain PAEs contributes, in addition to what has already been mentioned above, to the texture and consistency. The cosmetics in categories 2 and 3 therefore contain the highest amounts of PAEs. In category 4 and 5 cosmetics (nail polish and facial make-up, and eyeliners and eyeshadows, respectively), aroma fixation is not as critical as in the cosmetics in categories 1 and 2. PAE is added to nail polish (category 4) to prevent chipping and cracking of the product (by increasing the flexibility, consistency and durability of the polish). The percentage of positive samples in categories 4 and 5 was lower, with only 1.66 and 3.48% of positive samples, respectively.

**Figure 2:**
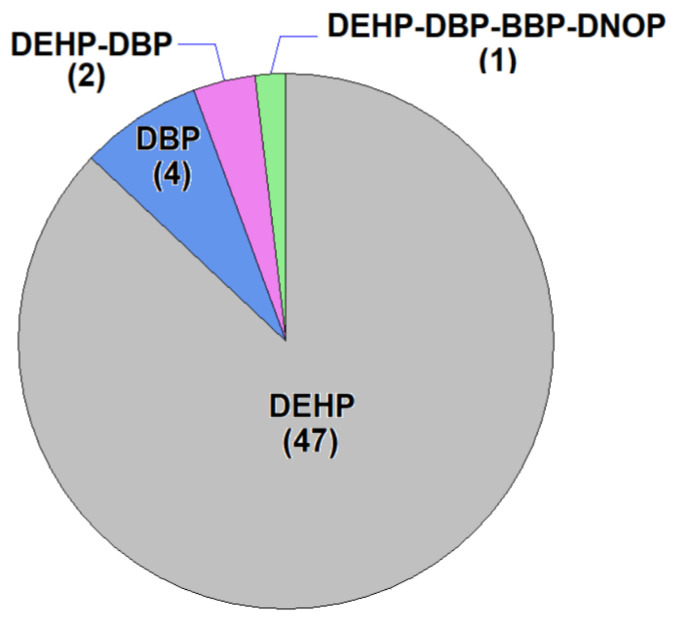
Pie chart of number of positive samples (*n* = 54) according to the type of phthalates detected (number of positive samples in parenthesis).

Regarding the number of PAEs per sample, most of the cosmetics analyzed exhibited a monophthalate character (See Figure 2). Indeed, only one phthalate was detected in 51 of the 54 positive samples (94.4%). In these cases, DEHP appeared in 47 samples, while only DBP was detected in the remaining four samples. The simultaneous occurrence of two PAEs was observed in two other samples in categories 4 and 5, in both cases DEHP and DBP. The final positive sample was a nail polish (category 4), in which four different phthalates were identified: BBP, DEHP, DNOP and DBP. In this case, the presence of the phthalates was probably due to contamination during an inappropriate production process. The remaining PAEs considered (DPP, DIPP, DMEP, DMP and PIPP) were not detected in any of the samples at concentrations higher than 1 µg mL^−1^. The number of phthalates detected in each sample according to the type of cosmetic is shown in Table 1. In summary, different PAEs were detected 59 times, in 54 positive samples from a total of 1110 cosmetics analyzed.

The concentrations of the different phthalates determined in positive samples in each of the cosmetic categories considered are summarized in Table 2. BBP and DNOP only appeared in a sample from category 4, together with DEHP and DBP, at concentrations in the range 1.50–2.70 µg mL^−1^. DBP was present in seven samples, at concentrations in the range 1.0 to 6.0 µg mL^−1^. In these cases, the low number of detections for these PAEs in all categories impedes establishment of any patterns. By contrast, DEHP was the most abundant PAE, being detected in all five categories of cosmetics analyzed.

The concentrations of DEHP in categories 3 and 5 were slightly higher than those in the other groups. Two samples in category 3 (a lipstick) and category 5 (a golden glitter eyeshadow) yielded very high concentrations of DEHP, probably (given the quantity reported) due to contamination of the sample in an inadequate production process, including poor fabrication practices, and/or use of contaminated packaging or raw materials.

## 3. Discussion

**Figure 3:**
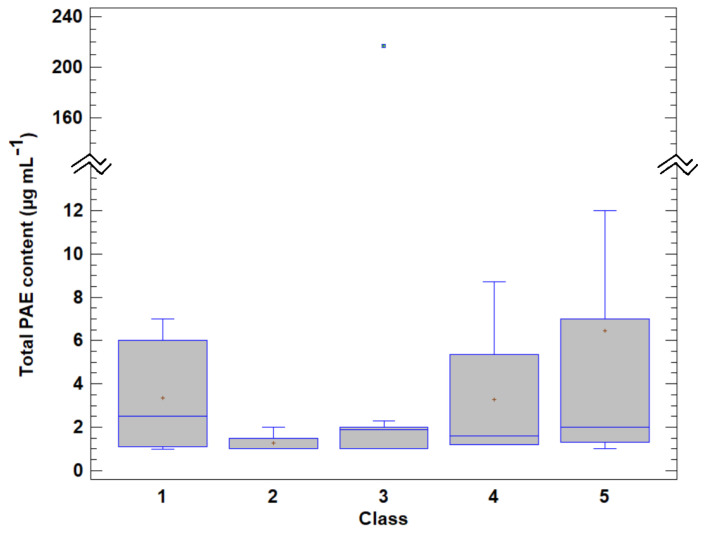
Box and whisker plot of the concentrations of DEHP according to the different categories of cosmetics considered in this study.

A box and whisker plot of the total concentration of PAEs according to the different categories considered in this study is shown in Figure 3. High total values were obtained in classes 1 and 5 due to the previously commented upon influence of high DEHP levels in these classes. However, the one-way ANOVA indicated no significant difference (*p* = 0.8912) between the mean of the total PAE contents in the different categories. An ANOVA was also used to assess the potential impact of the sample origin and the year of analysis on the PAE total content. The results again indicated no significant difference (*p* = 0.7987) in the concentration of phthalates in relation to the country of origin. Thus, the phthalate contents of the samples from Spain and France were similar. This result was expected, as the same regulatory framework regarding the use and concentration of phthalates in cosmetics is applied in Spain and France. It therefore appears that manufacturers in both countries comply with the same regulations, resulting in comparable levels of phthalates in their products. This also implies that the manufacturing processes used by cosmetic companies in Spain and France are comparable, ensuring uniform PAE content control according to the European regulations. These findings probably also apply to other European countries applying standard EC 1223/2009.

Regarding the year of production, if the highly contaminated lipstick sample from 2018 (with a DEHP content 217 µg mL^−1^) is considered an outlier, the results also indicate no significant difference (*p* = 0.1145) in the total PAE contents between the different years of the study period (2016–2020). This finding suggests that cosmetic manufacturers have maintained consistent formulations over the years, including the use of similar ingredients, production processes and manufacturing practices regarding phthalate content. Maintenance of the regulatory framework regarding PAEs in cosmetics from 2009 to the present has also led manufacturers to adjust their formulations accordingly, with the levels of phthalates in cosmetics remaining relatively stable. The consistency or stability in phthalate levels in cosmetics over the years indicates that regulatory efforts to ensure product safety have not decisively influenced industrial practices. Therefore, continuous monitoring and research is essential to evaluate any possible future effects or trends in phthalate contents in cosmetics that could arise due to implementation of the current legislative framework.

The results of the present five-year survey were compared (regarding the method of determination and the concentrations detected) with previously reported findings regarding PAEs in cosmetics.

In general, in order to determine PAEs in most cosmetics samples, an extraction step is required before the instrumental measurement for quantification (generally by HPLC-MS or GC-MS). Details of the extraction procedure and the determination technique used in the previously published papers are summarized in Table S1 in the Supplementary Materials. Different procedures were used to extract PAEs from cosmetics. Thus, liquid–liquid extraction with agitation or ultrasonic treatment followed (or not) by centrifugation was used with different solvents [11,16,17,18,19,20]; in other cases, extraction was carried out by solid phase extraction (SPE) with C18 [21,22], matrix solid-phase dispersion (MSPD) with florisil ([4,23] and this work) or solid phase micro-extraction (SPME) with polydimethylsiloxane-divinylbenzene (PDMS/DVB) fibers [24]. Mostafa and Shaaban [25] diluted perfume samples once with ethanol as a sample pretreatment.

In relation to the quantification technique, with the sole exception of Pérez-Fernández et al. (2013) [22], who used micellar electrokinetic chromatography (MEKC) with UV detection, the techniques most commonly used to determine PAEs in cosmetics are high-pressure liquid chromatography (HPLC) with different detectors [16,17,19,21,26] and gas chromatography–mass spectrometry (GC-MS) ([11,18,20,21,23,24,25] and this work). In all cases, the analytical figures of merit of the liquid and gas chromatographic methods used were sensitive enough to enable exact, precise determination of phthalate esters in samples of different types of cosmetics.

The concentrations of phthalate esters detected in cosmetics in other papers published in recent years are summarized in Table 3. There is a large difference in the quantity of samples assayed, the types of cosmetics analyzed, and also the number and characteristics of PAEs determined. In general, the concentrations of the diverse PAEs are detected in the µg g^−1^ or µg mL^−1^ range. In several published works, an inadequate use of significant figures has been observed in the presentation of the concentrations of the determined phthalates. In the present paper, the criterion applied was to cite the concentrations of PAEs obtained from other works by other authors in the same way as they appear in the original work, that is, with the same units and the same significant figures.

The highest levels are found in nail cosmetics. Orsi et al. [17] reported DEP, DBP and DEHP levels in nail polish and nail polish removers between 0.7 and 3.0% (*w*/*w*), which is equivalent to concentrations of 7000 to 30,000 µg g^−1^. Other authors also detected comparable high levels in this type of sample: Hubinger et al. [26] reported concentrations of DBP in nail enamel products of up to 122–62,607 µg g^−1^, and Koniecki et al. [11] documented levels of DBP in nail polishes in the range 3478–24,304 µg g^−1^. Mostafa and Shaaban [25] reported concentrations of DEP in perfumes in the range 0.35 up to 5766 µg mL^−1^. However, other researchers detected similar levels of PAEs in cosmetics to those reported in the present study, ranging between not detected up to several hundreds of µg g^−1^ or µg mL^−1^: Llompart et al. [23] in rinse-off and leave-on cosmetics (levels comprised between n.d. and 357 µg g^−1^), Kim et al. [20] in 100 cosmetic products from South Korea (n.d.–600 µg mL^−1^), or Hubinger et al. [26] in hair products, lotions, body washes, shampoos and creams (from <10 to 316 µg g^−1^). Overall, the variability in phthalate levels in cosmetics can be attributed to a combination of factors related to the type of cosmetic, formulation, regulation, manufacturing, storage and market dynamics. Different cosmetic formulations may contain different amounts of phthalates depending on the specific ingredients and manufacturing processes used. Some brands may include higher concentrations of some phthalates to enhance the characteristics of the product, while others may use lower concentrations or alternative ingredients. In addition, regulatory requirements impose limits on the concentration of phthalates. In the USA, European Union and other countries, the use of certain phthalates (such as DBP, DEHP and DEHP) is restricted, and cosmetics sold in some countries are therefore likely to have lower phthalate levels than those sold in countries with less stringent regulations. Moreover, the manufacturing process (raw materials, production methods and quality control measures, among others), as well as the product age and storage conditions can influence the concentration of phthalates in the final product. Finally, reformulation of these products by some brands to reduce or eliminate phthalates in response to consumer concerns or regulatory legislation is another possible cause of the variation observed.

In general, the phthalates most frequently used in cosmetic products (and which should therefore be the most commonly detected) are low-molecular-weight phthalates (LMW-PAEs) such as DBP, DEP and DMP (see, as an example, reference [23]). These LMW-PAEs are used because of their effectiveness as solvents in fragrances, helping to stabilize and even disperse the aroma. In addition, they help to enhance the texture of cosmetic items, facilitating smoother application and uniform distribution on the skin. Furthermore, LMW-PAEs tend to be more cost-effective and easily accessible than other phthalates, making them attractive for inclusion in cosmetic formulations.

The use of DEHP or other high-molecular-weight phthalates (HMW-PAE) in cosmetic products is not advisable because of the potential health risks associated with prolonged exposure, particularly when applied directly on the skin, as well as restrictive regulation on its use [1,27]. A clear example of this pattern of preponderance of LMW over HMW-PAEs is reported by Hubinger [26], who analyzed 84 different cosmetic products to determine the PAE content and found that DEP was the predominant phthalate, with levels ranging from not detected to 36,006 µg g^−1^ (except for nail enamel products in which DBP was the most abundant PAE, detected in the range 122 to 62,607 µg g^−1^). The concentrations of the remaining PAES, including DEHP, were below 10 µg g^−1^ in all cases. Koo and Lee [16] detected concentrations of DEP and DBP in perfumes, nail polish and deodorants in the range of thousands of µg mL^−1^. Similar results were reported by Koniecki et al. [11] for concentrations of these two LMW-PAEs, with maximum values of 25,542 µg g^−1^ for DEP. However, in this case, high concentrations of DEHP (an HMW-PAE) were also detected in fragrances (up to 521 µg g^−1^) and nail polishes (up to 1045 µg g^−1^). This is not an isolated situation, as DEHP has been detected and continues to be detected in numerous studies in which phthalates are measured in cosmetics. Other examples of the presence of high levels of DEHP together with LMW phthalates (as in the present study) can also be given. Orsi et al. [17] detected high concentrations of DEP and DBP in different nail cosmetics in the range 0.7–3.0% (*w*/*w*), although these authors also noted similar concentrations of DEHP of between 1.0 and 1.7% (*w*/*w*). Llompart et al. [23] detected DEP and DBP in ranges of 0.665–357 and 0.518–141 µg g^−1^, respectively, in different cosmetic samples of diverse origins; however, high concentrations of DEHP (0.578–25.8 µg g^−1^) were also reported in these studies. Moreover, Al-Saleh and Elkhatib [24] reported high concentrations of DEP in perfumes (maximum DEP 25,270 µg mL^−1^, and maximum DMP 420 µg mL^−1^); nevertheless, these authors also reported concentrations of DEHP up to 174 µg mL^−1^. In other recent work on colognes [28], higher concentrations of DEHP in eau de toilette and perfumes (n.d.–1785 µg mL^−1^) than in other LWM-PAEs (such as DBP and DMP) were detected. Finally, Mostafa and Shaaban [25] also detected DEHP in 95% of the 47 perfumes evaluated (in the range n.d. to 377.67 µg mL^−1^), in addition to other LNW phthalates. These authors attributed the presence of DEHP in cosmetics to potential contamination during manufacturing from containers containing DEHP.

**Table 3:** Concentrations of phthalate esters detected in cosmetics in different published papers.

Samples (*n*)	Origin of Samples	Phthalates Analyzed	Type of Sample (*n*)	Concentration (Mean or Range)	Ref.
Cosmetic and personal care products (*n* = 252)	Canada	DEP, DMP, DIBP, DBP, DEHP, BBP, DNOP, DMEP, DEEP, DOIP, DMPP, DPEP, DNHP, HEHP, DBEP, DCHP, DDCP, DUP (18)	Fragrances (*n* = 30)	DEP 0.6–25,542 µg g^−1^; DEHP 6.5–521 µg g^−1^	[11]
			Hair care products (*n* = 24)	DEP 0.8–1223 µg g^−1^	
			Deodorants (*n* = 31)	DEP 7.3–3634 µg g^−1^	
			Nail polishes (*n* = 20)	DIBP n.d.–0.4 µg g^−1^; DBP 3478–24,304 µg g^−1^; DEHP 3.8–1045 µg g^−1^	
			Body lotions and body creams (*n* = 29)	DEP 12–5549 µg g^−1^; DIBP n.d.–4.1 µg g^−1^	
			Skin cleansers (*n* = 20)	DEP 1.5–277 µg g^−1^; DIBP 0.6–1 µg g^−1^; DBP 1.8–6.6 µg g^−1^; DEHP n.d.–30 µg g^−1^	
			Baby products (oils, lotions, shampoos and diaper creams) (*n* = 98)	DEP 0.8–2566 µg g^−1^; DBP 0.9–1.8 µg g^−1^; DEHP n.d.–15 µg g^−1^	
Cosmetics products (*n* = 102)	Korea	DEHP, DEP, DBP, BBP (4)	Perfumes (*n* = 42)	DEHP 0.7 µg mL^−1^; DEP 3044 µg mL^−1^; DBP 444 µg mL^−1^; BBP 1.6 µg mL^−1^	[16]
			Nail polishes (*n* = 21)	DEHP 1.6 µg mL^−1^; DEP 1.6 µg mL^−1^; DBP 1671 µg mL^−1^	
			Hair products (*n* = 31)	DEP 3.3 µg mL^−1^	
			Deodorants (*n* = 8)	DEP 1473 µg mL^−1^	
Nail cosmetic products (*n* = 52)	European Union	DMP, DEP, DIBP, BBP, DBP, DEHP (6)	Nail lacquers (*n* = 27)	DEP 1.6–1.8 % (*w*/*w*); DBP 0.7–2.1 % (*w*/*w*); DEHP 0.3–1.4 % (*w*/*w*))	[17]
			Lacquer removers (*n* = 25)	DEP 2.0–3.0 % (*w*/*w*); DBP 1.0–1.5 % (*w*/*w*); DEHP 1.0–1.7 % (*w*/*w*)	
Personal care products (*n* = 170)	Albany, NY, USA	DMP, DEP, DBP, DIBP, BBP, DEHP, DNHP, DCHP, DNOP (9)	Rinse-off products (surfactant-based formulations such as shampoos, body washes and baby wash) (*n* = 41)	DMP 0.02 µg g^−1^; DEP 159 µg g^−1^; DIBP 0.04 µg g^−1^; DBP 0.05 µg g^−1^; DNHP 0.02 µg g^−1^; BBP 0.02 µg g^−1^; DEHP 0.50 µg g^−1^	[18]
			Leave-on products (*n* = 109)	DMP 0.59 µg g^−1^; DEP 389 µg g^−1^; DIBP 0.95 µg g^−1^; DBP 388 µg g^−1^; DNHP 0.38 µg g^−1^; BBP 0.80 µg g^−1^; DEHP 2.99 µg g^−1^	
			Baby care products (*n* = 20)	DMP 0.06 µg g^−1^; DEP 4.12 µg g^−1^; DIBP 0.01 µg g^−1^; DBP 0.02 µg g^−1^; BBP 0.01 µg g^−1^; DEHP 0.74 µg g^−1^	
Cosmetics samples (*n* = 57)	Shaanxi, China	DMP, DBP, DIDP (3)	Perfumes (*n* = 15)	Total PAE n.d.–68,377 µmol kg^−1^	[19]
			Nail polishes (*n* = 14)	Total PAE 88.9–65,999 µmol kg^−1^	
			Oily cosmetics (*n* = 11)	Total PAE n.d.–9038 µmol kg^−1^	
			Liquid skin care (*n* = 9)	Total PAE n.d.–78.0 µmol kg^−1^	
			Powder-based cosmetics (8)	Total PAE n.d.–83.1 µmol kg^−1^	
Cometic products (*n* = 100)	Suwon, South Korea	BBP, DBP, DEHP (3)	Body lotions (*n* = 10)	DEHP 0.76–2.50 µg mL^−1^	[20]
			Body washes (*n* = 10)	DEHP 0.5–5.20 µg mL^−1^	
			Face creams (*n* = 10)	DEHP 0.35–2.88 µg mL^−1^	
			Foam cleansing agents (*n* = 10)	DBP 2.00–5.70 µg mL^−1^; DEHP 0.80–1.70 µg mL^−1^	
			Hand creams (*n* = 10)	DEHP 0.57–1.60 µg mL^−1^	
			Manicures (*n* = 10)	BBP 2.40 µg mL^−1^; DEHP 0.20–0.71 µg mL^−1^	
			Perfumes (*n* = 30)	DBP 0.5 µg mL^−1^; DEHP 0.1–600.00 µg mL^−1^	
			Sun creams (*n* = 10)	DEHP 0.25–6.26 µg mL^−1^	
Cosmetic products (*n* = 15)	Random origin	DEP, DPP, DBP, BBP, DCHP, DEHP, DOP (7)	Hair sprays	BBP n.d.–1.33 mg kg^−1^; DBP n.d.–5289 mg kg^−1^; DCHP n.d.–36.26 mg kg^−1^; DEHP n.d.–115.2 mg kg^−1^	[21]
			Perfumes		
			Deodorants		
			Creams		
			Lotions		
Perfumes (*n* = 15)	Madrid Spain	DMP, DEP, DAP, DPP, DBP, DPP, DCP, BBP, DEHP (9)	Perfumes (*n* = 15)	DMP n.d.–1207 mg L^−1^; DEP n.d.-3115 mg L^−1^; DAP n.d.-520 mg L^−1^; DPP n.d.–331 mg L^−1^; DCP 557–1496 mg L^−1^	[22]
Cosmetic samples (*n* = 26)	International brands purchased in Spain	DMP, DEP, DIBP, DBP, DMEP, DIPP, DPP, BBP. DIHP, DEHP, DCHP, DPHP, DOP, DINP, DIDP (15)	Rinse-off products (shampoos, liquid soaps, hair conditioners and body scrub) (*n* = 7)	DEP 0.716–2.47 µg g^−1^; DIPP n.d.–0.154 µg g^−1^	[23]
			Leave-on products (body milks, moisturizing lotions, sun block, hands cream, anti-aging cream, aftershave and deodorants) (*n* = 19)	n.d.	
Perfumes (*n* = 47)	International brands from Saudi Arabia	DMP, DEP, DBP, BBP, DEHP (5)	Perfumes (*n* = 47)	DEP 0.220–25,270.370 µg mL^−1^; DMP 0.119–420.206 µg mL^−1^ DBP n.d.–0.6424 µg mL^−1^; BBP 0.006–201.724 µg mL^−1^; DEHP n.d.–174.786 µg mL^−1^	[24]
Perfumes (*n* = 40)	Saudi Arabia	DMP, DEP, DBP, BBP, DEHP (5)	Perfumes (*n* = 40)	DMP n.d.–60.00 µg mL^−1^; DEP 0.35–5766.00 µg mL^−1^; DBP n.d.–66.00 µg mL^−1^; BBP n.d.–22.50 µg mL^−1^; DEHP n.d.–377.67 µg mL^−1^	[25]
Adult-use and baby care cosmetic products (*n* = 84)	Washington, D.C., USA	DMP, DEP, BBP, DBP, DEHP (5)	Nail enamel products (*n* = 24)	DBP 122–62,607 µg g^−1^; Other PAEs < 10 µg g^−1^	[26]
			Antiperspirants or deodorants (*n* = 12)	DEP 164–2699 µg g^−1^; Other PAEs < 10 µg g^−1^	
			Perfumes (*n* = 11)	DEP 1328–36,006 µg g^−1^; Other PAEs < 10 µg g^−1^	
			Hair products (*n* = 7)	DEP 80–316 µg g^−1^; Other PAEs < 10 µg g^−1^	
			Lotions (*n* = 3)	DEP n.d.–133 µg g^−1^; Other PAEs < 10 µg g^−1^	
			Body washes (*n* = 2)	All PAEs < 10 µg g^−1^	
			Shampoo (*n* = 1)	All PAEs < 10 µg g^−1^	
			Baby shampoos and body washes (*n* = 13)	DEP 10–274 µg g^−1^; Other PAEs < 10 µg g^−1^	
			Baby creams, lotions and oils (*n* = 11)	DEP 114–234 µg g^−1^; Other PAEs < 10 µg g^−1^	
Colognes (*n* = 1147)	Spain and France	BBP, DEHP, DNOP, DPP, DBP, DIPP, DMEP, DMP, PIPP (9)	Eau de cologne (*n* = 41)	n.d.	[28]
			Eau de toilette (*n* = 789)	DEHP n.d.–500 µg mL^−1^; DBP n.d.–7 µg mL^−1^; DMEP n.d.–6 µg mL^−1^; DMP n.d.–2 µg mL^−1^	
			Fragrance (*n* = 87)	DEHP n.d.–20 µg mL^−1^; DMP n.d.–20 µg mL^−1^	
			Perfume (*n* = 230)	DEHP n.d.–1785 µg mL^−1^; DBP n.d.–21 µg mL^−1^; DMEP n.d.–7 µg mL^−1^; DMP n.d.–2 µg mL^−1^	
Cosmetic products (*n* = 1110)	France and Spain	BBP, DEHP, DNOP, DPP, DBP, DIPP, DMEP, DMP, PIPP (9)	Soaps, shampoos, shower gels and related products (*n* = 154)	DEHP n.d.–7.00 µg mL^−1^; DBP n.d.–1.00 µg mL^−1^	[This Work]
			Creams, hand and body lotions (*n* = 109)	DEHP n.d.–2.00 µg mL^−1^	
			Gloss and lipsticks (*n* = 290)	DEHP n.d.–217 µg mL^−1^; DBP n.d.–1.50 µg mL^−1^	
			Nail polish (*n* = 241)	BBP n.d.–1.50 µg mL^−1^; DEHP n.d.–2.80 µg mL^−1^; DBP n.d.–1.70 µg mL^−1^; DNOP n.d.–2.70 µg mL^−1^	
			Facial make-up and skin cosmetics (*n* = 316)	DEHP n.d.–38.0 µg mL^−1^; DBP n.d.–6.00 µg mL^−1^	

Thus, the prevalence of DEHP as one of the commonly detected phthalates in the analyzed cosmetics raises significant concerns. Despite being prohibited for inclusion in cosmetics products by legislation in the European Union, USA and other countries, DEHP continues to be detected and has two potential origins: (i) its use as a plasticizer to enhance the flexibility and durability of cosmetics, and (ii) as a result of possible contamination of cosmetic products from plastic films and plastic containers containing DEHP. As DEHP remains present in cosmetics (although concentrations have decreased since its restriction) and given the known associated health risks, such as endocrine disruption and reproductive toxicity [29], the widespread presence of this HMW-PAE in cosmetics raises alarms regarding potential consumer exposure. This highlights the need for continued control and regulation, emphasizing the importance of monitoring phthalate levels in personal care products. Authorities and regulatory bodies should consider implementing stricter controls and regulations regarding phthalate levels in such products.

Other phthalates have already been used or will undoubtedly be used in the coming years to replace the currently regulated compounds, with the simple strategy of modifying the alcohol chain radicals. More than ten years ago in 2012, Dodson et al. [30] pointed out this tendency after an analysis of different “conventional” and “alternative” commercial vinyl and fragrance products that could potentially contain PAEs. In the example of vinyl, DEHP was detected at concentrations of up to 28%, while in the second group of fragrance/perfume compounds, concentrations of up to 1.4% DEP were reported. Another three phthalates have also been detected in various products: DCP, DINP and DPP. These three compounds have obviously been used to replace the existing, regulated phthalates (DBP, BBP and DEHP among others); however, the novel compounds have endocrine disruption properties of similar (DCP) or lower (DINP and DPP) potency than the previously used compounds. Aldegunde-Louzao et al. (2023) [31] detected the same trend in a seven-year study on textile products, in which it is evident that DEHP and DBP have been partially replaced by DINP. More recently, Lee and Choi [32] pointed out that DEHP has been replaced by DINP and DIDP, which are also increasingly used as plasticizers. These authors indicate that, considering current exposure levels, DINP will cause endocrine disruption in a similar way to DEHP. Furthermore, in relation to DIDP, these authors emphasized that the limited information available about this phthalate prevents correct evaluation of the effects. Thus, exposure to these other emerging phthalates must be assessed in detail because the compounds may have similar toxicities to already banned compounds.

## 4. Materials and Methods

### 4.1. Sample Collection

In the present study, 1110 different cosmetics samples were analyzed in a specialized quality control laboratory for textile and cosmetic products during the period 2016–2020. The samples were classified into five different categories. Category 1 (*n* = 154) included soaps, shampoos, shower gels and related products. Category 2 (*n* = 109) comprised creams, hand and body lotions. Category 3 (*n* = 290) comprised gloss and lipsticks. Category 4 (*n* = 241) consisted of nail polish samples. Finally, Category 5 (*n* = 316) included all facial make-up and skin cosmetics (including eyeliners, eyeshadows, make-up bases, etc.). The samples were supplied directly by manufacturers or large suppliers in their original, correctly sealed containers. A small number of pre-production samples, provided in sterile containers (not the final container), were also tested. All samples were preserved in darkness at a controlled temperature until analysis, which was generally carried out within 48 h of receipt. Most of the samples were of Spanish origin (*n* = 1005), and the others (*n* = 105) were from France.

### 4.2. Reagents and Apparatus

All solvents used in this study were of chromatographic grade (obtained from Merck, Darmstadt, Germany). Ultrapure water was obtained from a Milli-Q purification system (Millipore, Bedford, MA, USA). Standards for the nine phthalate esters (BBP, DEHP, DNOP, DPP, DBP, DIPP, DMEP, DMP and PIPP) were obtained from the LGC Group (Middlesex, UK), Sigma-Aldrich (Madrid, Spain) and Scharlab (Barcelona, Spain). Deuterated di-*n*-octyl phthalate (DNOP-d4) and deuterated bis-(2-ethylhexyl) phthalate (DEHP-d4) were used as internal standards in the chromatographic analysis and were supplied, along with deuterated benzyl butyl phthalate (BBP-d4), used as a surrogate, by Laboratorios CIFGA S.A. (Lugo, Spain). Anthracene-d10, also used as an internal standard, was obtained from Sigma-Aldrich (Vallensbaek Strand, Denmark). GC-MS/MS analysis of the nine phthalates was conducted with a gas chromatograph Agilent model 7890B (Agilent Technologies, Santa Clara, CA, USA) to an Agilent 7000 C tandem mass spectrometer (Agilent Technologies, Santa Clara, CA, USA). Statistical analysis was carried out with Statgraphics Centurion XVIII v.18.1.12 (Statistical Graphics Corporation, Rockville, MD, USA).

### 4.3. Analytical Procedure

The samples were prepared according to the procedure described by Celeiro et al. and Llompart et al. [4,23] with minor modifications. Briefly, 0.1 g aliquots of samples were weighed out and treated with 0.2 g of anhydrous sodium sulphate, before 0.4 g of florisil and 20 µL of the BBP-d4 surrogate internal standard (50 mg L^−1^) were added. The mixture was homogenized and passed through a florisil microcolumn. The analytes were eluted with ethyl acetate, and the extract was filtered through a 0.45 µm filter. Ten µL of internal standard (50 mg L^−1^) was added to the solution obtained, which was made up to the final volume in a 1 mL volumetric flask. This solution was subjected to the GC-MS/MS determination procedure described below.

Phthalates in different samples are commonly determined by GC-MS [2,33]. For the cosmetics in the present study, measurements were carried out following the European Standard 16521:2014 [34] with a few modifications. The detailed working conditions for analysis were reported in a previous paper [28]. Briefly, the analytes from the prepared samples were evaporated at high temperatures in the gas chromatograph inlet and placed in the chromatographic column. The analytes were separated on a fused silica capillary column (HP-5MS, 30 m × 0.25 mm ID 0.25 μm). The separated analytes were then placed in the electron impact ion source of the mass spectrometer, and the ions generated were transferred to the mass spectrometer analyzer, where they were selected on the basis of the *m*/*z* ratio and quantified using a detector. Signal acquisition by the GC-MS/MS equipment was conducted in multiple reaction monitoring (MRM) mode. The experimental conditions and temperature gradient program, the retention time for each phthalate and internal standard, and the qualification and quantification transitions are summarized in Table 4.

The analytes were identified by a comparison of the samples with external calibration standards, based on the relative signal obtained for the selected characteristic ions using MRM detection mode. The analytes were quantified by external calibration, with signal correction by means of internal standards. Following this procedure, the previously indicated nine phthalate esters were quantified in all samples of the five categories of cosmetics considered. The analytical measurements were subjected to a stringent quality control process throughout the measurement period to ensure the reliability of the results. In addition, our laboratory participated (with satisfactory results) in international interlaboratory exercises aimed at testing proficiency in determining phthalates in textile and related products organized by the Institute for Interlaboratory Studies, The Netherlands (Starink, 2022) [35]. In the case at hand, satisfactory analytical figures of merit were achieved. Data from the validation of the applied analytical method are presented in Table S2 in the Supplementary Materials.

## 5. Conclusions

Increased consumer awareness and advocacy for safer cosmetic products, along with clear and strict regulatory standards, have led the cosmetics industry to prioritize the reduction in or removal of phthalates from their formulations. The present paper reports the results of a five-year quality control system aimed at verifying the phthalate content of a wide range of cosmetics samples from Spain and France. The following conclusions can be drawn from these results and their comparison with other previously published findings: (i) Gas chromatography combined with tandem mass spectrometry has proven to be a suitable analytical technique for the measurement of phthalates in cosmetic samples. Furthermore, the gradual replacement of banned phthalates by unregulated ones (which can also have negative effects on human health) indicates the need to develop analytical methods capable of detecting all phthalates, regardless of the type of R and R’ chains included. This objective can be achieved using GC-MS/MS procedures based on precursor ion scanning. Therefore, in addition to the advantages of detection by tandem MS, in this specific case, the possibility of detecting any PAE present in the cosmetic is the great and main advantage of GC-MS/MS in relation to other different techniques such as those indicated in Table S1 (MECK-UV, HPLC-DAD and GC-MS). (ii) Non-compliant samples (in which the total phthalate content exceeded 1 µg mL^−1^) represented 4.86% of the total of 1110 samples analyzed. Although the proportion of positive samples is relatively low, this figure is important as these batches are removed from the chain and must undergo reprocessing (or be discarded) before entering the market, which generates costs that could be avoided by better management of the production process. (iii) DEHP is the most frequently occurring phthalate, followed by DBP. No significant differences were found in the phthalate contents of the samples in relation to the country of origin (France or Spain) or to the year of sampling. (iv) Other studies in which PAE concentrations in cosmetics were determined reported a large variety of formulations and concerns, as in the present study. (v) Although LMM-PAEs are generally preferably used in cosmetic formulations, in the present and many previous studies, high concentrations of HMM-PAEs (especially DEHP) have been detected. (vi) The notable persistent presence of DEHP in many formulations indicates the need for stricter control of this type of product.

Therefore, the control of phthalates in cosmetic products is essential to ensure safe use, adherence to legal regulations and maintenance of product quality and also to promote transparency and informed use to consumers.

## Figures and Tables

**Table 1:** Summary of the PAE content in the analyzed samples according to the category considered.

Category			1	2	3	4	5	Total
Number of samples analyzed			154	109	290	241	316	1110
Number of positive samples *			6	12	21	4	11	54
% positive samples in the category			3.89	11.01	7.24	1.66	3.48	---
Amount of PAEs detected	Amount of PAEs	PAEs						
1 PAE	51	DEHP	5	12	20	2	8	47
		DBP	1	---	1	---	2	4
2 PAEs	2	DEHP	---	---	---	1	1	2
		DBP	---	---	---	1	1	2
4 PAEs	1	BBP	---	---	---	1	---	1
		DEHP	---	---	---	1	---	1
		DNOP	---	---	---	1	---	1
		DBP	---	---	---	1	---	1
Total samples	54			Total detections				59

* [Total PAE] > 1 µg mL^−1^.

**Table 2:** Distribution of the number and type of PAEs in the positive samples. All concentrations are given in µg mL^−1^.

PAE	Category	1	2	3	4	5
BBP	Positive samples	---	---	---	1	
	Mean	---	---	---	1.50	
	Median	---	---	---	1.50	
	Range	---	---	---	---	
DEHP	Positive samples	5	12	20	4	9
	Mean	3.80	1.25	12.4	1.55	6.92
	Median	3.0	1.00	1.95	1.20	2.00
	Range	1.00–7.00	1.00–2.00	1.00–217	1.00–2.80	1.00–38.0
DBP	Positive samples	1	---	1	2	3
	Mean	1.00	---	1.50	1.35	2.90
	Median	1.00	---	1.50	1.35	2.90
	Range	---	---	---	1.00–1.70	1.00–6.00
DNOP	Positive samples	---	---	---	1	
	Mean	---	---	---	2.70	
	Median	---	---	---	2.70	
	Range	---	---	---	---	

**Table 4:** GC-MS/MS experimental conditions. Quantitation and confirmation of transitions for the analyzed PAEs and internal standards. Reprinted from Ref. [28].

Chromatographic Conditions			
Injection volume		1.0 µL	
Carrier gas		1 mL min^−1^, Helium (constant flow)	
Injection mode		Split 10:1–Split flow: 10 mL min^−1^	
Inlet temperature		290 °C	
Temperature ramp		Initial: 50 °C (hold 1 min) Ramp 1: 50 °C to 190 °C (20 °C min^−1^) Ramp 2: 190 °C to 220 °C (8 °C min^−1^) Ramp 3: 220 °C to 310 °C (30 °C min^−1^, hold on 2 min)	
Total time		16.75 min	
MSD temperature		280 °C	
MSD mode		Electronic impact (−70 eV)	
Acquisition mode		MRM	
MS Quad temperature		150 °C (max: 200 °C)	
MS Source temperature		230 °C (max: 250 °C)	
**Analyte**	**Retention** **Time (min)**	**Quantification** **Transition (*m*/*z*)**	**Qualification** **Transition (*m*/*z*)**
BBP	14.22	206 → 149	149 → 65; 149 → 93
DEHP	14.88	279 → 149	167 → 149; 279 → 93
DNOP	15.51	279 → 149	279 → 93
DPP	13.25	237.2 → 149	149 → 65; 149 → 93;49 → 121
DBP	11.98	223 → 149	205 → 149
DIPP	12.75	237.2 → 149	149 → 65; 149 → 93; 149 → 121
DMEP	12.30	176 → 149	207 → 149
DMP	8.36	163 → 77	163 → 133
PIPP	13.00	237.2 → 149	149 → 65; 149 → 93; 149 → 121
Anthracene-d10	10.68	188 → 160	188 → 186
DEHP-d4	14.87	171 → 153	153 → 69
DNOP-d4	15.45	283 → 153	283 → 125; 283 → 97
BBP-d4	14.90	153 → 69	153 → 97

## Data Availability

The raw data supporting the conclusions of this article will be made available by the authors on request.

## Supplementary Materials

The following supporting information can be downloaded at https://www.mdpi.com/article/10.3390/molecules29204823/s1, Table S1: Characteristics of the analytical methods employed in the different works for the quantification of PAEs; Table S2: Data validation results of the GC-MS/MS method for the determination of phthalates in cosmetic samples.
