# Supplementary material for: Phthalate Esters in Different Types of Cosmetic Products: A Five-Year Quality Control Survey
Source: Molecules. 2024 Oct 11;29(20):4823. doi: 10.3390/molecules29204823 (PMC11510284; doi:10.3390/molecules29204823)
Supplement: Supplementary file 1 [file molecules-29-04823-s001.zip › molecules-3227488-supplementary.pdf]

Table S1. Characteristics of the analytical methods employed in the different works for the quantification of PAEs.

Sample	Determined phthalates	LOD/LOQ	Extraction procedure	Quantification Technique	Reference
Cosmetic and personal care products (n=252)	DEP, DMP, DIBP, DBP, DEHP, BBP, DNOP, DMEP, DEEP, DOIP, DMPP, DPEP, DNHP, HEHP, DBEP, DCHP, DDCP, DUP (18)	DMP 0.5 µg g^{-1}<br>DEP 0.5 µg g^{-1}<br>DIBP 0.1 µg g^{-1}<br>DBP 0.1 µg g^{-1}<br>DEHP 0.5 µg g^{-1}	0.4 g of sample was accurately weighed into a 15 mL test tube. 40 mg aliquots of three internal standards, DMP-d4, DBP-d4 and DEHP-d4 were spiked into each sample. For lotions, skin cleansers, shampoos, and hair gels, two different solvent systems were used in order to obtain high recoveries of both short chain (DMP and DBP) and long chain (DEHP) phthalates. Mechanical shaking and centrifugation were also used to enhance the extraction process. At the end of extraction, the upper clear organic solvent layer was collected and diluted 10 times prior to instrumental analysis.	GC-MS	[11]
Cosmetics products (n=102)	DEHP, DEP, DBP, BBP (4)	DEHP 0.004 µg mL^{-1}<br>DEP 0.0005 µg mL^{-1}<br>DBP 0.0005 µg mL^{-1}<br>BBP 0.0005 µg mL^{-1}	0.1 g of sample was spiked with 2500 ng mL^{-1} of DNHP as internal standard. This mixture was added to 10 mL methanol, vortexed, and centrifuged (3000 rpm for 15 min)	HPLC (detector not detailed)	[16]
Nail cosmetic products (n=52)	DMP, DEP, DIBP, BBP, DBP, DEHP (6)	DMP 0.4 µg mL^{-1}<br>DEP 0.4 µg mL^{-1}<br>DIBP 0.5 µg mL^{-1}<br>BBP 0.5 µg mL^{-1}<br>DBP 0.5 µg mL^{-1}<br>DEHP 0.6 µg mL^{-1}	Aliquots (0.100 g) of the cosmetics sample were diluted with ethanol-water 90:10, and ultrasonicated for 15mm at 40 ◦C. After centrifugation, an aliquot of the clear supernatant was collected, filtered and injected in the chromatograph.	HPLC-DAD	[17]
Personal care products (PCPs) (n=170)	DMP, DEP, DBP, DIBP, BBP, DEHP, DNHP, DCHP, DNOP (9)	DMP 2 ng g^{-1}<br>DEP 2 ng g^{-1}<br>DBP 2 ng g^{-1}<br>DIBP 2 ng g^{-1}<br>BBP 2 ng g^{-1}<br>DEHP 2 ng g^{-1}<br>DNHP 2 ng g^{-1}	Samples (0.05 g) were extracted in a 12 mL glass tube. After fortification of samples with 500 ng each of d4- labeled phthalate internal standards, 2 mL of Milli-Q water was added and equilibrated overnight at room temperature. Samples were then extracted twice with 4 mL aliquots of methyl tert-butyl ether (MTBE) by shaking in an orbital shaker for 30 min, followed by centrifugation at 4400 g for 20 min. The combined extracts were divided into two equal halves and concentrated under a gentle	GC-MS	[18]

		DCHP 2 ng g^{-1}<br>DNOP 10 ng g^{-1}	stream of nitrogen; the solvent was reconstituted with hexane for analysis of phthalates by gas chromatography.		
Cosmetics simples (n=57)	DMP, DBP, DIDP (3)	LOD 5.1 µmol kg^{-1}<br>LOQ 9.2 µmol kg^{-1}<br>(Total PAEs determined phthalic acid)	Samples (0.2 g) were weighed in a 25 mL glass vial followed by the addition of 1 mL n-hexane. 50 µL of 168 µmol L^{-1} PA-d4 (IS), 1 mL of ethanol, and 8 mL of 8 mol L^{-1} KOH. The vial was capped and heated at 80 °C with magnetic stirring for 15 min at 800 rpm. After cooling, the upper n-hexane phase and colored middle layer were collected and discarded. Ten mL of 6 mol L^{-1} HCl was then added, and the solution was mixed and allowed to cool to ambient temperature. For liquid phase micro-extraction, 8 mL of the acid hydrolysate was transferred to a 10 mL polypropylene tube containing 600 µL of TBP. The tube was shaken for 2 min, and the resulting uniformly distributed cloudy liquid was centrifuged at 6000 rpm for 3 min. The upper TBP phase (620 ± 20 µL) was then transferred to a 1.5 mL vial and mixed with an equal volume of methanol:water (4:1, v/v). After filtration through a 0.45-µm membrane, the sample was subjected to HPLC-MS/MS.	HPLC-MS/MS	[19]
Cosmetic products (n=100)	BBP, DBP, DEHP (3)	BBP 0.0006 µg mL^{-1}<br>DBP 0.0005 µg mL^{-1}<br>DEHP 0.0007 µg mL^{-1}	Cosmetics samples were prepared by adding 1 g of each cosmetic to the hexane/acetone mixture and adjusting the volume to 10 mL. Thereafter, samples were dispersed for 1 h using an ultrasonicator and centrifuged at 3000 rpm for 10 min, and 5 mL of the supernatant was accurately retrieved. One mL of the internal standard solution was added to the supernatant and the hexane/acetone mixture was added to reach a final volume of 10 mL, which was used as the cosmetic solution for analysis.	GC-MS	[20]
Cometic products (n=15)	DEP, DPP, DBP, BBP, DCHP, DEHP, DOP (7)	10.0 - 100.0 µg kg^{-1}	Sonication-assisted extraction with methanol and clean-up with C18 SPE.	HPLC-DAD and GC-MS	[21]
Perfumes (n=15)	DMP, DEP, DAP, DPP, DBP, DPP, DCP, BBP, DEHP (9)	DMP 8.6 mg L^{-1}<br>DEP 8.6 mg L^{-1}<br>DAP 7.6 mg L^{-1}<br>DPP 7.8 mg L^{-1}<br>DBP 11.4 mg L^{-1}	Five hundred µL of perfume was transferred in a glass tube and 10 mL of methanol was added following by sonication during 30 min. The sample was then evaporated to dryness and redissolved in 25 mL 40% (v/v) methanol. Solid phase extraction (SPE) with a C18 cartridge was used for clean-up of the sample. The cartridge was	MEKC-UV	[22]

		DPP 7.1 mg L^{-1}<br>DCP 15.6 mg L^{-1}<br>BBP 10 mg L^{-1}<br>DEHP 19.2 mg L^{-1}	conditioned with 5 mL methanol, 5 mL water and 5 mL 40% (v/v) methanol. The sample was loaded onto the column at a slow rate and after loading, the column was washed with 5 mL 40% (v/v) methanol. Finally, phthalates were eluted with 5 mL of methanol and injected into the micellar electrokinetic chromatography system.		
Real cosmetic samples, in both rinse-off and leave-on cosmetic formulations (n=26)	DMP, DEP, DIBP, DBP, DMEP, DIPP, DPP, BBP, DIHP, DEHP, DCHP, DPHP, DOP, DINP, DIDP (15)	DMP 0.0025 µg g^{-1}<br>DEP 0.053 µg g^{-1}<br>DIBP 0.081 µg g^{-1}<br>DBP 0.080 µg g^{-1}<br>DMEP 0.0081 µg g^{-1}<br>DIPP 0.0069 µg g^{-1}<br>DPP 0.0016 µg g^{-1}<br>BBP 0.0054 µg g^{-1}<br>DIHP 0.050 µg g^{-1}<br>DEHP 0.082 µg g^{-1}<br>DCHP 0.0060 µg g^{-1}<br>DPHP 0.0014 µg g^{-1}<br>DOP 0.0051 µg g^{-1}<br>DINP 0.12 µg g^{-1}<br>DIDP 0.30 µg g^{-1}	0.1 g of sample was weighed into a 10 mL glass vial and spiked with 10 µL of DEHP-d4 surrogate solution (2.5 µg mL^{-1} in acetone). The sample was gently blended with 0.2 g of a drying agent (anhydrous Na_2SO_4) and 0.4 g of dispersing sorbent (Florisil) in a porcelain mortar with a porcelain pestle until a homogeneous mixture was obtained (ca. 5 min). The mixture was transferred to a glass Pasteur pipette (approximately 150 mm), with a small amount of glass wool at the bottom, containing 0.1 g of Florisil (to obtain a further degree of fractionation and sample clean-up). The samples were eluted by gravity flow with 1mL ethyl acetate or hexane/acetone (1:1, v/v), collecting 1 mL of extract into a volumetric flask.	GC-MS	[23]
Perfumes (n=47)	DMP, DEP, DBP, BBP, DEHP (5)	DEP 0.513 µg L^{-1}<br>DMP 0.514 µg L^{-1}<br>DBP 0.770 µg L^{-1}<br>BBP 0.455 µg L^{-1}<br>DEHP 0.629 µg L^{-1}	Samples were diluted 1:10 with deionized water. For each analysis, 10 mL of the diluted perfume sample was spiked with 6 µL of internal standard (2 µg L^{-1} of deuterated di-n-propylphthalate-3,4,5,6-d4,) and placed in a 20-mL glass vial. The samples were mixed well and then left to equilibrate for 10 min at 40 °C. The sample vial was then moved to the CombiPAL autosampler agitator. SPME was conducted at 90 °C for 13 min with shaking at 500 rpm. The analytes were thermally desorbed from the SPME fiber into the GC-MS inlet.	GC-MS	[24]
Perfumes (n=40)	DMP, DEP, DBP, BBP, DEHP (5)	DMP 1.9 10^{-3} µg mL^{-1}<br>DEP 1.5$10^{-3}$ µg mL^{-1}<br>DBP 1.2 10^{-3} µg mL^{-1}	Perfumes were diluted in ethanol (1:5).<br>1 µL was used for GC-MS analysis.	GC-MS	[25]

		BBP 2.1 10^{-3} µg mL^{-1}<br>DEHP 1.0 10^{-3} µg mL^{-1}			
Adult-use and baby-care cosmetic products (n=84)	DMP, DEP, BBP, DBP, DEHP (5)	LOQ in the range 1 to 10 µg g^{-1}	Approximately 1 g of each test portion was weighed in a 40 mL beaker, mixed with about 3 g of Celite, and transferred to a 15 mL extraction tube. The sample/Celite mixture was covered with a filter disk and was compacted firmly with a stirring rod, and hexane was then passed through the filter disk by gravity flow. The extract was collected in a 10 mL volumetric flask. For solid deodorants, the sample was first dispersed in hexane to create slurry and then extracted as described above.	HPLC-UV	[26]
Colognes (n=1147)	BBP, DEHP, DNOP, DPP, DBP, DIPP, DMEP, DMP, PIPP (9)	LOD in the range 0.004 to 0.015 µg mL^{-1}	1.0 mL of the liquid sample was diluted with ethyl acetate to a total volume of 10 mL in a volumetric flask. The resulting solution was then filtered through a 0.45 µm syringe filter. Subsequently, 980 µL of this sample solution was mixed with 20 µL of a 50 mg L^{-1} internal standard working solution and injected into the GC-MS/MS system.	GC-MS/MS	[29]

Table S2. Data validation results of the GC-MS/MS method for the determination of phthalates in cosmetic samples.

PAE	Sensitivity		Accuracy**			Reproducibility***			
	LOD* (µg mL^{-1})	LOQ* (µg mL^{-1})	Recovery (%)			Concentration level 0.1 µg mL^{-1} (except for DPP and DIPP: 0.05 µg mL^{-1})		Concentration level 0.3 µg mL^{-1} (except for DPP and DIPP: 0.15 µg mL^{-1})	
			Medium	Max	Min	Standard deviation	RSD (%)	Standard deviation	RSD (%)
DMP	2.0 10^{-3}	6.8 10^{-3}	93.84	99.28	90.54	2.9 10^{-3}	2.93	4.9 10^{-3}	1.65
PIPP	6.6 10^{-3}	22 10^{-3}	92.89	111.4	74.90	2.6 10^{-3}	2.59	5.4 10^{-3}	1.80
DPP	9.1 10^{-3}	31 10^{-3}	93.60	111.2	74.81	1.9 10^{-3}	3.90	2.4 10^{-3}	1.58
DBP	0.3 10^{-3}	1.0 10^{-3}	102.4	121.2	90.33	4.2 10^{-3}	4.21	5.4 10^{-3}	1.80
DMEP	8.5 10^{-3}	28 10^{-3}	86.52	112.1	77.90	4.4 10^{-3}	4.37	15 10^{-3}	5.04
DIPP	5.3 10^{-3}	18 10^{-3}	93.34	112.4	75.56	1.7 10^{-3}	3.36	2.6 10^{-3}	1.73
BBP	12 10^{-3}	39 10^{-3}	92.38	110.4	83.48	2.8 10^{-3}	2.78	6.1 10^{-3}	2.03
DEHP	49 10^{-3}	163 10^{-3}	113.0	139.1	91.43	3.4 10^{-3}	3.44	5.2 10^{-3}	1.75
DNOP	43 10^{-3}	142 10^{-3}	89.85	116.7	67.46	5.6 10^{-3}	5.62	5.5 10^{-3}	1.84

*: Limit of detection (LOD) and Limit of quantification (LOQ) were calculated as 3 and 10 times the standard deviation of the blank.

**: Accuracy was studied by means of spike recovery method.

***: Reproducibility was calculated from the results of the same samples (at two different concentration levels) analyzed in nine different days by different analysts.
